# RACK-1 Acts with Rac GTPase Signaling and UNC-115/abLIM in *Caenorhabditis elegans* Axon Pathfinding and Cell Migration

**DOI:** 10.1371/journal.pgen.1001215

**Published:** 2010-11-18

**Authors:** Rafael S. Demarco, Erik A. Lundquist

**Affiliations:** Programs in Genetics and Molecular, Cellular, and Developmental Biology, Department of Molecular Biosciences, University of Kansas, Lawrence, Kansas, United States of America; University of California San Diego, United States of America

## Abstract

Migrating cells and growth cones extend lamellipodial and filopodial protrusions that are required for outgrowth and guidance. The mechanisms of cytoskeletal regulation that underlie cell and growth cone migration are of much interest to developmental biologists. Previous studies have shown that the Arp2/3 complex and UNC-115/abLIM act redundantly to mediate growth cone lamellipodia and filopodia formation and axon pathfinding. While much is known about the regulation of Arp2/3, less is known about regulators of UNC-115/abLIM. Here we show that the *Caenorhabditis elegans* counterpart of the Receptor for Activated C Kinase (RACK-1) interacts physically with the actin-binding protein UNC-115/abLIM and that RACK-1 is required for axon pathfinding. Genetic interactions indicate that RACK-1 acts cell-autonomously in the UNC-115/abLIM pathway in axon pathfinding and lamellipodia and filopodia formation, downstream of the CED-10/Rac GTPase and in parallel to MIG-2/RhoG. Furthermore, we show that RACK-1 is involved in migration of the gonadal distal tip cells and that the signaling pathways involved in this process might be distinct from those involved in axon pathfinding. In sum, these studies pinpoint RACK-1 as a component of a novel signaling pathway involving Rac GTPases and UNC-115/abLIM and suggest that RACK-1 might be involved in the regulation of the actin cytoskeleton and lamellipodia and filopodia formation in migrating cells and growth cones.

## Author Summary

In the developing nervous system, the growth cone guides axons of neurons to their correct targets in the organism. The growth cone is a highly dynamic specialization at the tip of the axon that senses cues and responds by crawling toward its target, leaving the axon behind. Key to growth cone motility are dynamic cellular protrusions called lamellipodia and filopodia. These protrusions are required for growth cone movement and steering. The genes that are involved in lamellipodia and filopodia formation in the growth cone are still being discovered, and studies to understand how these genes act together in cell signaling events that control growth cone movement are in their infancy. Here we report discovery of a new gene necessary for growth cone movement in *Caenorhabditis elegans* called *rack-1*. This gene is conserved in vertebrates and is involved in cellular signaling. We show that it interacts in a novel manner with other cell signaling genes (the *Rac GTPase* genes) and a gene involved in lamellipodia and filopodia formation, called *unc-115/abLIM*. We think that *rack-1* is involved in a novel cellular signaling event involving Rac GTPases that regulates lamellipodia and filopodia protrusion in the growth cone during nervous system development.

## Introduction

The actin cytoskeleton is necessary for the formation of cellular protrusions, lamellipodia and filopodia, that underlie morphogenetic events such as cell migration and axon pathfinding [1]–[4]. Unraveling the complex molecular events that regulate actin structure and dynamics in migrating cells and growth cones will be central to understanding the development of multicellular organisms and the nervous system in particular. Migrating cells and growth cones display dynamic lamellipodial and filopodial protrusions consisting of a meshwork of actin filaments and bundles of actin filaments, respectively [4]–[8]. Lamellipodia and filopodia serve to guide cells and growth cones and also provide in part the motile force necessary for cell migration and growth cone advance [9]. A complex interplay of filopodial and lamellipodial dynamics controlled by guidance receptors and their ligands is the basis for guidance outgrowth and migration.

In cultured cells, the actin-nucleating Arp2/3 complex controls the formation of lamellipodial networks [1], [10], [11], whereas the anti-capping protein Enabled controls filopodial formation [12], [13]. Enabled also affects axon pathfinding in *Caenorhabditis elegans* [14], [15]. In migrating growth cones in *C. elegans*, the Arp2/3 complex is required for both lamellipodial and filopodial formation [16], likely due to the contribution of Arp2/3-nucleated actin filaments to filopodial bundles [7]. The actin-binding protein UNC-115/abLIM [17] also controls lamellipodial and filopodial formation in *C. elegans* growth cones [16], and acts in parallel to the Arp2/3 complex in axon pathfinding [16]–[18], indicating that UNC-115/abLIM may be contributing to both lamellipodial and filopodial formation in growth cones. The signaling pathways that control Arp2/3 activation are well documented. The Arp2/3 activators WASP and WAVE act downstream of Cdc42 and Rac GTPases respectively to regulate Arp2/3 activity [11], [19]–[22]. In *C. elegans* axon pathfinding, WVE-1/WAVE acts downstream of CED-10/Rac and WSP-1/WASP acts downstream of the MIG-2/RhoG GTPase to regulate Arp2/3 [18].

While much is known about the Arp2/3 signaling pathway, less is known about the control of UNC-115/abLIM in lamellipodia and filopodia formation. The conserved UNC-115/abLIM proteins have multiple LIM domains at the N terminus and an actin-binding villin headpiece domain at the C terminus [17], [23], [24]. The central region of the molecule contains a short region of similarity shared with the dematin protein, which also contains a C terminal actin-binding villin headpiece domain. Previous studies in *C. elegans* showed that UNC-115/abLIM acts downstream of the CED-10/Rac GTPase in neuronal lamellipodia and filopodia formation [25]. The conserved seven-WD repeat molecule SWAN-1 physically interacts with the UNC-115 LIM domains and with Rac GTPases, and is normally required to attenuate Rac GTPase signaling [26], indicating that SWAN-1 might be a link between Rac signaling and UNC-115/abLIM.

A two-hybrid screen with the central region of UNC-115 identified the *C. elegans* Receptor for Activated C Kinase molecule (Rack1), called RACK-1 in *C. elegans* [27], [28]. Rack1 molecules are composed of seven WD repeats, which form a seven-bladed beta propeller structure that serves as a scaffold for protein-protein interactions [29]. Rack1 was first identified as a molecule that bound to activated protein kinase C and mediated its plasma membrane translocation [30], [31]. Further studies have shown that Rack1 acts with a very diverse set of signaling complexes and can mediate their sub cellular distributions and shuttling (reviewed in [32]). This diversity of interaction leads to a diversity of function for Rack1, including transcriptional and translational regulation, regulation of membrane trafficking, regulation of signal transduction, and cell adhesion [32]. Interestingly, Rack1 controls cell motility via its interaction with the Src tyrosine kinase [31], [33]. Rack1 is a substrate for Src tyrosine phosphorylation and acts as a repressor of Src in response to active PKC [31], [34]–[37]. Rack1 inhibits Src-induced cell motility in cultured 3T3 fibroblasts, and inhibits Src phosphorylation of p190RhoGAP [38], a modulator of Rho GTPase signaling and actin organization. Rack1 is also phosphorylated on tyrosine 52 by c-Abl, which is involved in Rack1 regulation of focal adhesion kinase and integrin function [39]. In *C. elegans*, RACK-1 has been shown to be involved in embryonic cytokinesis [27]. *C. elegans* RACK-1 regulates membrane trafficking and recycling endosome distribution via interaction with dynactin, and thus might regulate the microtubule motor dynein. As a consequence, *rack-1* loss of function leads to defects in cytokinesis and chromosome separation in the early embryo.

Here we show that RACK-1 interacts with the actin-binding protein UNC-115/abLIM, and that RACK-1 is required for axon pathfinding. Genetic interactions indicate that RACK-1 acts in the UNC-115/abLIM pathway in axon pathfinding, downstream of the CED-10/Rac GTPase and in parallel to MIG-2/RhoG and the UNC-34/Enabled. Neuron-specific expression of RACK-1 is sufficient to rescue the axon pathfinding defects of *rack-1* mutants, indicating that RACK-1 acts cell autonomously in axon pathfinding. Furthermore, we show that RACK-1 is involved in migration of the gonadal distal tip cells, and that the signaling pathways involved in this process might be distinct from those involved in axon pathfinding. In sum, these studies pinpoint RACK-1 as a component of a signaling pathway involving Rac GTPases and UNC-115/abLIM, and suggest that RACK-1 might be involved in the regulation of the actin cytoskeleton and lamellipodia and filopodia formation in migrating cells and growth cones.

## Results

### RACK-1 interacts with UNC-115/abLIM in a yeast two-hybrid screen

**Figure 1:**
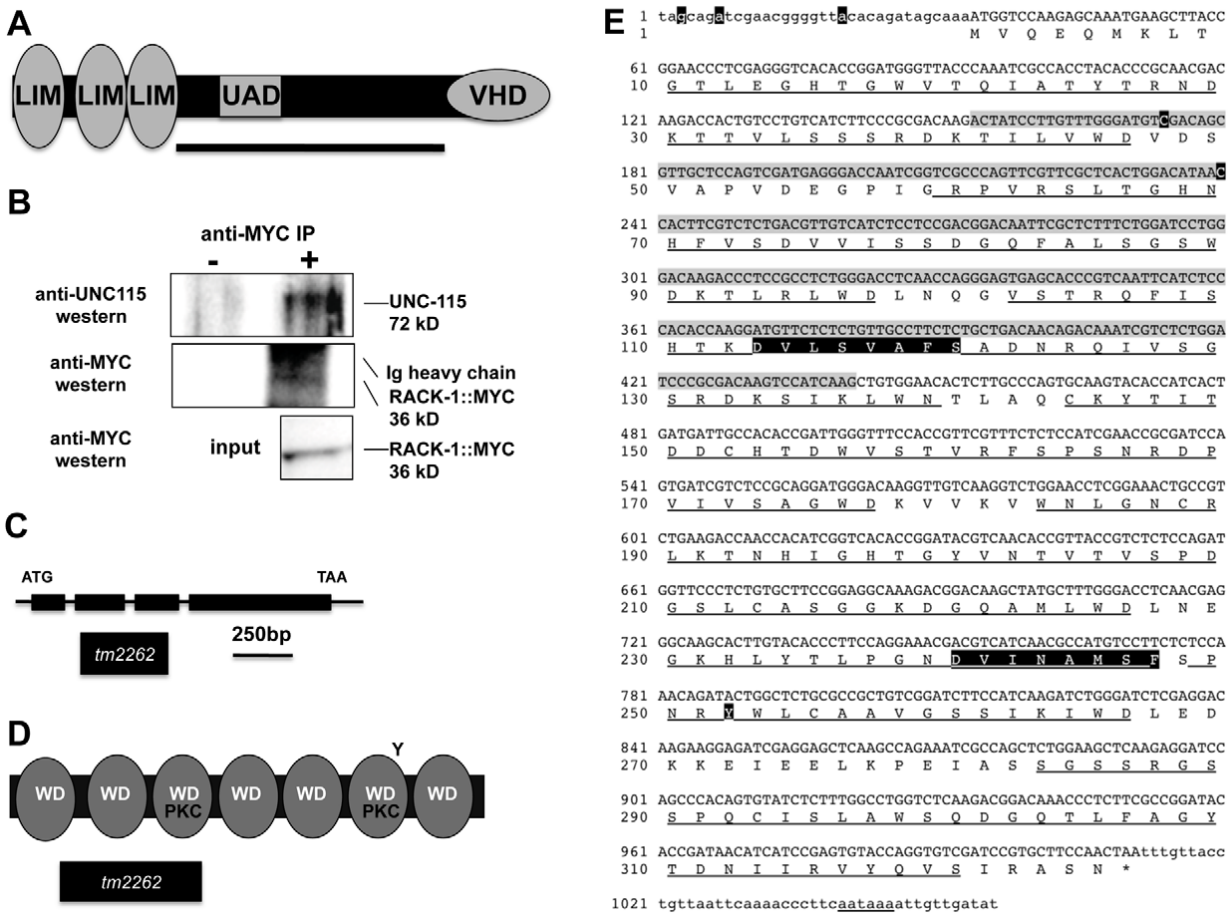
RACK-1 and UNC-115 interact by two-hybrid and co-immunoprecipitation. A) A schematic diagram of the 639-residue UNC-115 molecule. LIM = LIM domains; VHD  =  villin headpiece domain; UAD  =  unc-115/abLIM/dematin domain. The bar represents the region of the molecule used as bait in a two-hybrid screen. B) UNC-115 co-immunoprecipitated with MYC-tagged RACK-1. Lysates of worms expressing MYC-tagged RACK-1 were used in immunoprecipitation experiments with and without anti-Myc antibody. Western blots using anti-UNC-115 antibody [26] (top) showed that UNC-115 co-immunoprecipitated with RACK-1::MYC. Western blots using anti-Myc showed that RACK-1::MYC was expressed (input) and was immunoprecipitated. RACK-1::MYC ran just below the Ig heavy chain of the anti-Myc antibody used in the immunoprecipitation. C) The *rack-1* gene and *tm2262* allele. This model is based on the K07D7.1 gene model in Wormbase and on the cDNAs sequenced from the two-hybrid screen. *tm2262* is a 331-bp deletion. (D) Schematic diagram of the 325-residue RACK-1 protein. WD  =  WD repeat; PKC  =  conserved protein kinase C interaction sites; Y =  conserved tyrosine phosphorylated by Src in mammalian RACK. The region removed by the *tm2262* deletion is indicated. E) Sequence of the *rack-1* cDNA and protein. cDNA sequence: The sequence of the RACK-1 cDNA is based upon sequencing seven cDNAs isolated from the two-hybrid screen and on cDNA sequences in Wormbase. The predicted open reading frame is upper case, and the 5′ and 3′ untranslated regions are lower case. A predicted poly-A addition site is underlined, and poly A residues are present in the cDNAs at the end of the sequence shown here. The nucleotides removed by the *tm2262* deletion are shaded in grey. The starting points of the five independent cDNAs isolated in the two-hybrid screen are highlighted in black. Amino acid sequence: The predicted amino acid sequence is shown below the cDNA sequence. The WD repeats are underlined; the two conserved PKC interaction sites are highlighted in black; and the conserved tyrosine residue that is phosphorylated by Src in mammalian RACK is highlighted in black.

The actin-binding protein UNC-115/abLIM has three LIM domains in the N-terminus, a villin headpiece domain (VHD) in the C-terminus, and a middle region with unknown function that contains a highly conserved region across species, the UAD domain (UNC-115, abLIM, dematin) (Figure 1A) [17]. The VHD physically interacts with F-actin [24], [25], while the LIM domains are thought to mediate protein-protein interactions. Previous studies showed that the seven WD-repeat protein SWAN-1, a negative regulator of UNC-115 activity, interacts with the LIM domains of UNC-115 [26].

In order to identify molecules that interact physically with the non-LIM-domain region UNC-115, the central region of UNC-115 (residues 243 to 553 of the F09B9.2b molecule as described on Wormbase) was used as bait in a yeast two-hybrid screen (Figure 1A). This two-hybrid screen was performed at the Molecular Interaction Facility at the University of Wisconsin-Madison. The screen involved activation of β-galactosidase activity and *HIS5* expression in a liquid-based microtiter screening procedure (see Materials and Methods). From a total of 36 million *C. elegans* poly-A primed cDNAs screened, seven cDNAs that corresponded to the K04D7.1 gene (as annotated on Wormbase) were found. All seven cDNAs were found to activate when retested, and all seven cDNAs displayed bait-dependence and did not activate in the absence of the UNC-115 bait (data not shown).

The seven cDNAs represented five independent isolates (i.e. represented five different 5′ ends), with two of the isolates having two representatives each (Figure 1E). Three of the cDNAs contained the entire predicted K04D7.1 open reading frame, and two were missing some of the predicted 5′ open reading frame. All five cDNAs were in frame to the GAL4 activation domain in the pACT two-hybrid vector.

The K04D7.1 cDNAs were predicted to encode a molecule similar to vertebrate Receptor for Activated C Kinase (Rack1), called RACK-1 in *C. elegans* (Figure 1D and 1E) [27]. RACK-1 is predicted to contain seven WD repeats that form a seven-bladed beta-propeller similar to the beta subunit of G proteins [40]. Rack1 molecules define a conserved family of seven-WD repeat proteins, and are distinct from other families such as Gβ and AN11/SWAN-1 [26], [40]. Rack1 molecules are defined by two conserved regions that interact with protein kinase C, a conserved tyrosine residue that is phosphorylated by the Src tyrosine kinase, and a tyrosine residue at position 52 that is phosphorylated by c-Abl. The PKC interaction sites and the Src phosphorylated tyrosine are conserved in the *C. elegans* RACK-1 protein, but the c-Abl phosphorylated tyrosine at position 52 in human Rack1 is not present in *C. elegans* RACK-1 (Figure 1E). Two of the cDNAs isolated in the two-hybrid screen were missing coding region for the first predicted WD repeat and one of them was missing part of the second predicted WD repeat (Figure 1E).

### UNC-115 co-immunoprecipitated with RACK-1

To confirm that RACK-1 and UNC-115 interact in a complex, we determined if RACK-1 and UNC-115 co-immunoprecipitated from *C. elegans* extracts. We generated a transgene expressing MYC-tagged RACK-1 under its endogenous promoter and made animals transgenic for this construct. This transgene produced functional RACK-1::MYC, as it rescued the sterility, gonadal distal tip cell migration defects, and axon pathfinding defects caused by the *rack-1(tm2262)* deletion (see below). We immunoprecipitated MYC-tagged RACK-1 (RACK-1::MYC) using an anti-MYC antibody from animals harboring a *rack-1::myc* integrated gene (see Materials and Methods). Using anti-MYC western blots, we found that RACK-1::MYC (36 kD) was expressed in *C. elegans* extracts and that it was immunoprecipitated by this treatment (Figure 1B). Western blots using anti-UNC-115 antibody [26] showed the specific co-immunoprecipitation of UNC-115 (72 kD) with RACK-1::MYC (Figure 1B). In the absence of the anti-MYC antibody, RACK-1::MYC did not precipitate, and neither did UNC-115 (Figure 1B). Furthermore, we could detect no UNC-115 when extracts from *C. elegans* not expressing RACK-1::MYC were immunoprecipitated with the MYC antibody (data not shown). We repeated this co-immunoprecipitation two additional times, and the results of one representative experiment are shown in Figure 1.

### *rack-1* is required for axon pathfinding

*C. elegans* RACK-1 is a 325 amino-acid protein that has two regions similar to the PKC binding sites of vertebrate RACK and a conserved tyrosine that is phosphorylated by Src in vertebrate RACK. *C. elegans* PKC and Src isoforms are expressed in the nervous system, and both PKC and Src have been implicated in growth cone pathfinding and cell migration [41], [42]. Furthermore, we show above that RACK-1 interacts with UNC-115, a molecule that controls axon pathfinding in *C. elegans* [17]. Thus, we determined if RACK-1 was also involved in axon pathfinding in *C. elegans*.

**Figure 2:**
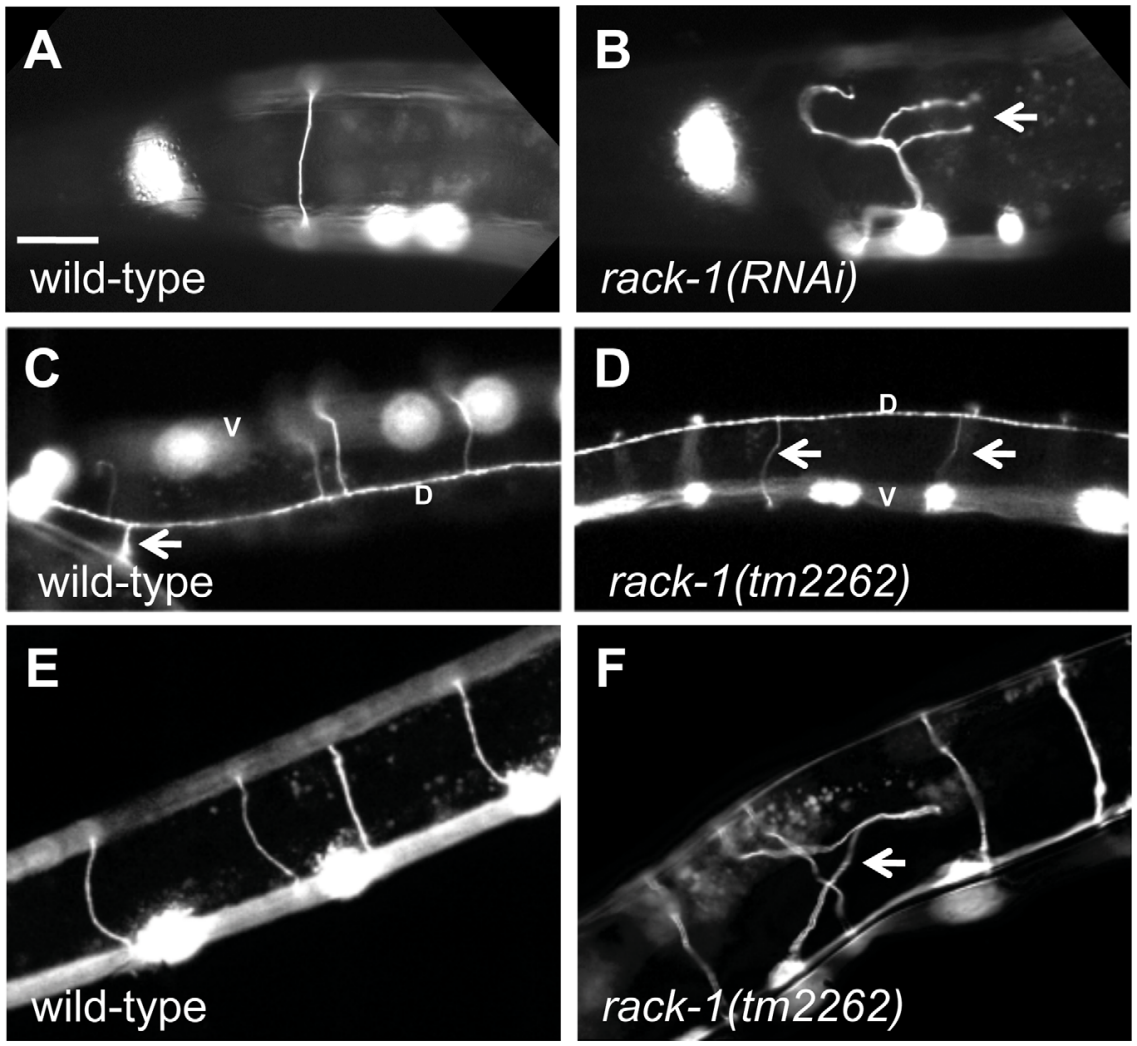
RACK-1 is required for VD/DD motor axon pathfinding. All panels are micrographs of animals with *unc-25promoter::gfp* expression (*juIs76* transgene) in the VD/DD GABAergic motor neurons. The scale bar in A represents 10 µm for all panels. (A and B) Wild type and *rack-1(RNAi)* showing the DD1/VD2 commissure on the left side of the animal. Misrouted and branched axons are indicated by an arrow. Dorsal is up, and anterior is left. (C and D) Dorsal views of the dorsal nerve cords (marked by a D) of wild type and *rack-1(tm2262)*. This dorsal view allows for demonstration of the sidedness of axon pathfinding. The cell bodies in the ventral nerve cord are out of focus (marked by a V). The arrow in C points to the DD1/VD2 commissure on the left side of the animal. All other VD/DD commissures go up the right side. Arrows in D point to VD/DD commissures that aberrantly extend up the left side of the animal in *rack-1(tm2262)*. (E and F) VD/DD commissures in wild type and *rack-1(tm2262)*. The arrow in F points to a misguided axon.

The VD and DD motor neurons are GABAergic neurons that control the coordination and movement of the nematode [43], [44]. The VD and DD cell bodies reside on the right side of the ventral nerve cord. Axons extend anteriorly, branch, and extend dorsally to form axon commissures (Figure 2). Upon reaching the dorsal cord, the axons branch again and extend posteriorly and anteriorly. We used an *unc-25::gfp* transgene (*juIs76*) to image the VD/DD neurons and their axons [45]. *unc-115(ky275*) disrupts axon pathfinding in these neurons, yielding in an uncoordinated movement phenotype [17]. We perturbed *rack-1* function using RNAi by injection (see Materials and Methods). In 22% of injected animals (n>100), *rack-1(RNAi)* disrupted the proper pathfinding of the VD and DD commissural axons (Figure 2A and 2B). The defects seen, such as axon misguidance, branching and premature termination, resembled the defects observed in *unc-115(ky275)* [17] and were never observed in wild-type animals. These results suggest that RACK-1 might be involved in axon pathfinding, similar to UNC-115.

A deletion of the *rack-1* locus, called *tm2262*, was isolated and kindly provided by the National Bioresource Project for the Experimental Animal “Nematode C. elegans” (S. Mitani). The *tm2262* deletion was an in-frame deletion that removed part of the first WD repeat, all of the second, and most of the third, including the predicted PKC interaction site in WD3 (Figure 1C–1E). Since *tm2262* is an in-frame deletion, *tm2262* animals might still produce truncated RACK-1 protein and *rack-1(tm2262)* might be a hypomorph. However, RNAi did not worsen the low brood size or axon defects of *rack-1(tm2262)* (see below; data not shown), indicating that it might be a strong loss of function allele.

**Figure 3:**
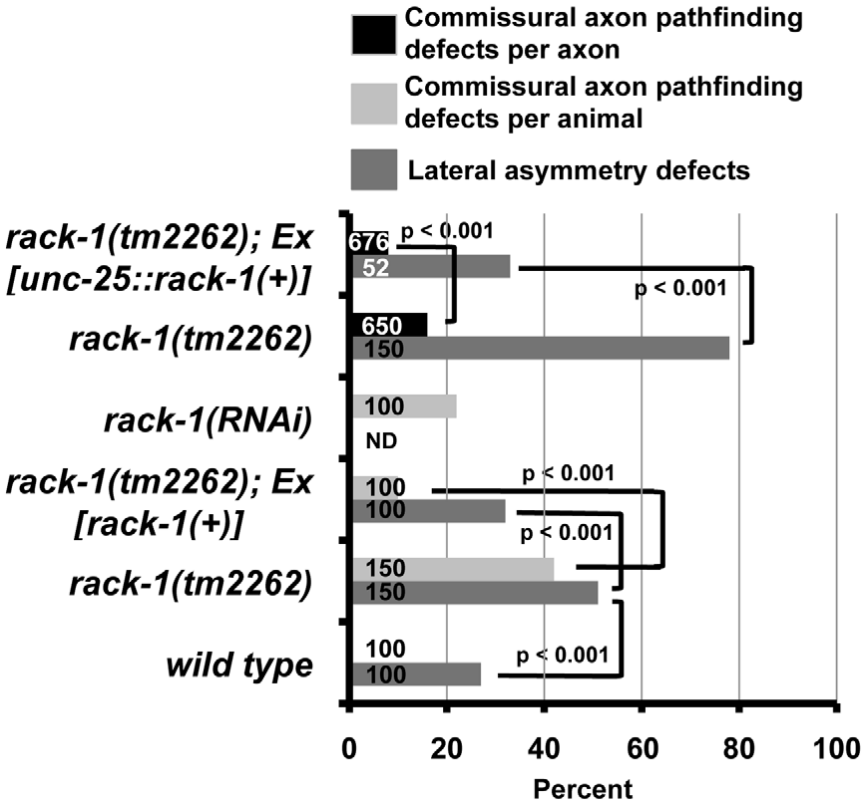
RACK-1 acts cell-autonomously in VD/DD motor axon pathfinding. The Y axis denotes genotype, and the X axis represents percentage of defects of the sort described in the legend. *Ex[rack-1(+)]* is the full-length *rack-1::myc* transgene under the *rack-1* endogenous promoter, and *Ex[unc-25::rack-1(+)]* is a transgene with *rack-1::gfp* expression driven by the *unc-25* promoter specifically in the GABAergic neurons, including the VD/DDs. VD/DD commissural axon pathfinding defects were scored by animal (the percent of animals with defective axons) or by axon (the percent of defect axons). Number of animals or axons scored is indicated in the bars. P-values for significance were determined by Fisher Exact Analysis.

Similar to RNAi of *rack-1*, the deletion allele *rack-1(tm2262)* caused pathfinding defects in the VD and DD motor neurons (Figure 2). Normally, all VD/DD commissures extend on the right side of the animal except DD1/VD2, which form a single commissure in the anterior (arrow in Figure 2C). *rack-1(tm2262)* displayed VD/DD commissures aberrantly extending up the left side of the animal (Figure 1D), and VD/DD axons that were misguided on their dorsal migrations (Figure 1D). In our hands, 27% of wild type animals harboring the *unc-25:gfp* transgene *juIs76* had VD/DD commissures on the left side in addition to DD1/VD2. However, 60% of *rack-1(tm2262); juIs76* showed VD/DD commissures on the left side (p<0.001) (Figure 3). In *juIs76* animals, generally only one or two left-side VD/DD were observed, whereas multiple axons on the left side were often observed in *rack-1(tm2262); juIs76* animals (Figure 2D). In addition, 42% of *rack-1(tm2262); juIs76* animals displayed VD/DD axon guidance and outgrowth defects such as axonal wandering, branching or termination (Figure 3), whereas *juIs76* alone showed no strong defects but did display some minor axon wandering.

To ensure that the axon guidance defects observed in *rack-1(tm2262)* were due to *rack-1* perturbation and not a background mutation, we rescued the VD/DD axon defects with a *rack-1::myc* transgene. *rack-1::myc* rescued both left-right defects and commissural guidance defects (60% to 32% (p<0.001) and 42% to 10% (p<0.001)) (Figure 3). Together, these results indicate that RACK-1 is required for VD/DD axon pathfinding.

### *rack-1(tm2262)* caused reduced brood size

*rack-1(tm2262)* animals were slow growing and had very low brood size. In a progeny count, ten wild type and ten *rack-1(tm2262)* animals were individually plated and then transferred to a new plate every day until egg laying ceased. The number of viable adult progeny resulting from each animal were counted and averaged. The average progeny count for a wild-type N2 animal was of 278.4 (s.d. = 32.72), while for *rack-1(tm2262)* the count dropped to 23.3 (s.d. = 9.9) (p<0.0001). A transgene containing the *rack-1* gene under its native promoter fused to the *gfp* coding region (*rack-1::gfp*) (see Materials and Methods) increased brood size in *rack-1(tm2262)* animals to 73.78 (s.d. = 17.18) (p<0.0001), suggesting that *rack-1::gfp* was functional and could rescue the brood size defect in *rack-1* animals. *rack-1::myc* could also rescue the low brood size of *rack(tm2262)* (data not shown). Thus, the reduction in brood size was due to *rack-1* and not due to genetic background in the *tm2262* strain.

The reduced brood size of *rack-1(tm2262)* seems to be predominantly due to decreased production of fertilized embryos. *rack-1* might affect oogenesis or spermatogenesis, but the nature of this sterility has not been explored. Previous studies indicate that *rack-1* also affects embryogenesis by regulating membrane trafficking and recycling endosome distribution via interaction with dynactin to control cytokinesis and chromosome separation in the early embryo [27].

### *rack-1::gfp* was expressed in most tissues, including neurons and the distal tip cells

**Figure 4:**
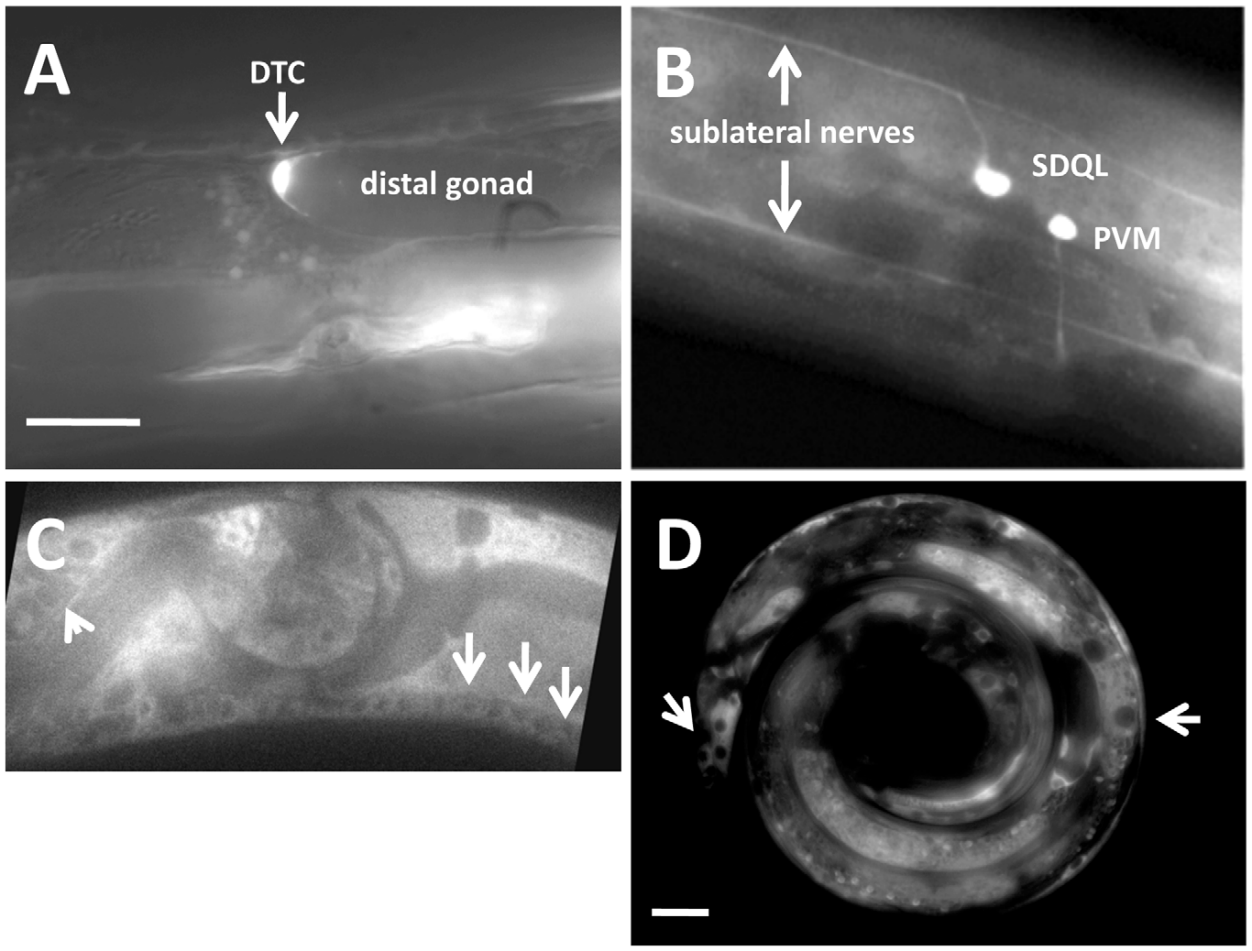
RACK-1 is expressed in most cells, including neurons and the gonadal distal tip cells. Panels are fluorescent micrographs of animals harboring *rack-1::gfp* transgenes. (A and B) are fusions of the *rack-1* promoter to *gfp*; and (C and D) are fusions of the entire full-length *rack-1* coding region to GFP. A) A distal tip cell expressed *rack-1::gfp*. B) Neurons expressed *rack-1::gfp*. C) Full-length RACK-1::GFP was expressed in neurons in the ventral nerve cord (arrows) and in the amphid (arrowhead). D) Full-length RACK-1::GFP was excluded from nuclei in tail hypodermis and gut (arrows). The scale bar in A represents 5 µm for A–C, and the scale bar in D represents 5 µm.

In order to determine where the *rack-1* gene is expressed, we constructed a reporter transgene consisting of the promoter region of *rack-1* fused to the *gfp* coding region (see Materials and Methods). *rack-1 promoter::gfp* was expressed in most if not all tissues. Due to mitotic loss of the transgene-bearing extrachromosomal array, we were able to analyze *rack-1 promoter::gfp* expression in mosaic animals in which we could discern specific cell types. *rack-1 promoter::gfp* was expressed in neurons as well as the distal tip cells of the gonad (Figure 4A and 4B).

**Figure 5:**
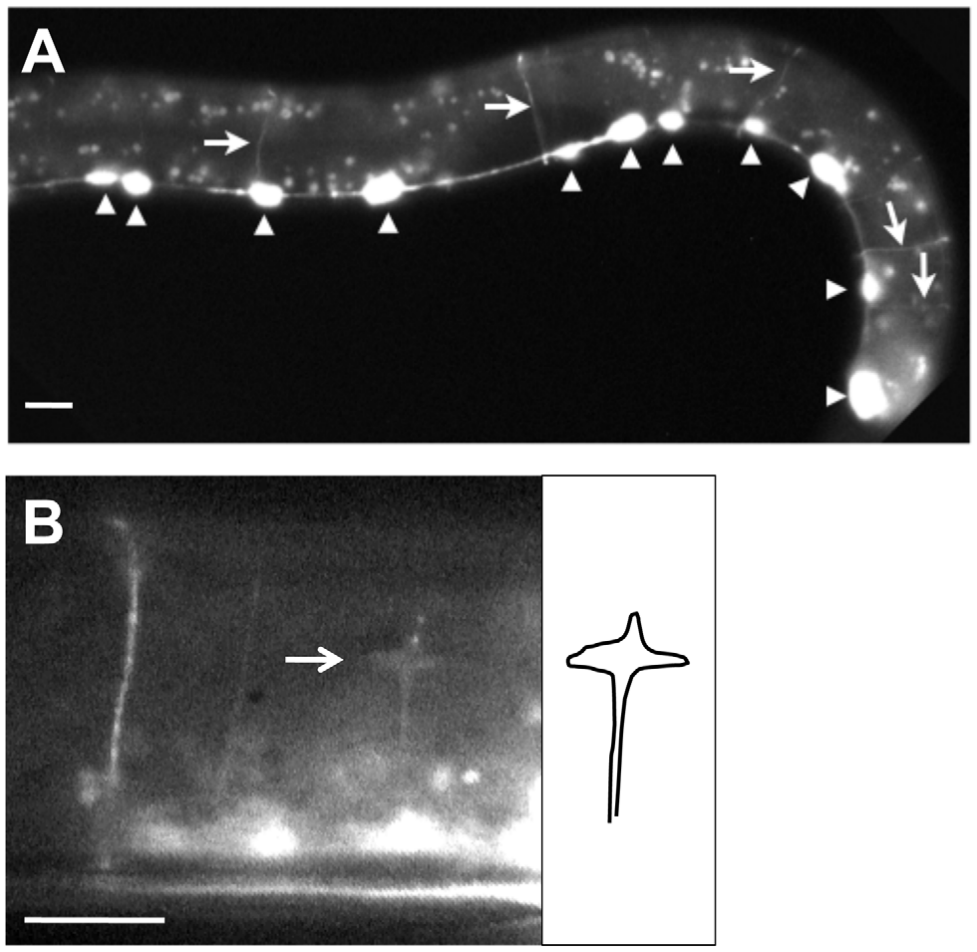
RACK-1::GFP expression in GABAergic motor neurons. Micrographs are of animals harboring an *unc-25::rack-1::gfp* transgene that expresses a full-length RACK-1::GFP fusion protein specifically in the VD/DD GABAergic motor neurons. A) RACK-1::GFP expression is specific to the VD/DD motor neurons. Arrowheads indicate cell bodies in the ventral nerve cord, and arrows indicate commissural axons. The punctate fluorescence throughout the animal is autofluorescence of the gut. B) RACK-1::GFP accumulated in a VD growth cone in an early L2 animal (arrow). The diagram at the right traces the outline of the growth cone and axon. The scale bars represent 5 µm.

In order to determine the subcellular localization of RACK-1 protein, we constructed a full-length *rack-1::gfp* fusion. This transgene is predicted to encode a full-length RACK-1 protein with GFP at the C-terminus (RACK-1::GFP). *rack-1::gfp* rescued the sterility and gonadal distal tip cell migration defects of *rack-1(tm2262)* mutants. RACK-1::GFP was present in the cytoplasm of cells and showed little if any nuclear accumulation (Figure 4C and 4D), although low levels of RACK-1::GFP in the nucleus cannot be excluded. RACK-1::GFP was present in the growth cones of extending VD commissural axons, but was present in the axons and cell bodies as well (Figure 5A and 5B).

### *rack-1* acts cell-autonomously in the VD/DD neurons to control axon pathfinding

*rack-1* was expressed in most if not all tissues in the animal, including neurons. To determine if RACK-1 is required in the VD/DD neurons themselves for axon pathfinding, we drove expression of *rack-1::gfp* specifically in the VD/DD neurons using the GABAergic neuron-specific *unc-25* promoter. The wild-type *rack-1(+)* coding region lacking the upstream promoter region was fused to *gfp* downstream of the *unc-25* promoter. The *Ex[unc-25 promoter::rack-1::gfp]* transgene was expressed specifically in the GABAergic neurons including the VD/DD neurons and nowhere else (Figure 5A). This transgene did not rescue the fertility defects and DTC migration defects of *rack-1(tm2262)* as did the genomic *rack-1(+)* transgene (data not shown), indicating that expression was specific to the VD/DD neurons. *Ex[unc-25 promoter::rack-1(+)]* rescued the lateral asymmetry defects and axon wandering defects of *rack-1(tm2262)* animals (Figure 3) (60% to 33% for lateral asymmetry defects and 16% to 8% for axon wandering defects; p<0.001 in both cases). In this experiment, individual axons were scored, due to the mosaic nature of the *Ex[unc-25 promoter::rack-1(+)]* transgene. These data indicate that *rack-1* acts cell autonomously in neurons in axon pathfinding.

### *rack-1(tm2262)* enhanced *mig-2(mu28)* and *unc-34(e951)*, but not *unc-115(ky275)* or *ced-10(n1993)*, in PDE axon pathfinding

The above results show that RACK-1 physically interacted with UNC-115/abLIM and that *rack-1* loss of function caused axon pathfinding defects similar to *unc-115*. Previous studies showed that UNC-115/abLIM acts downstream of the Rac GTPase CED-10/Rac and in parallel to MIG-2/RhoG in axon pathfinding [25], [46]. We next set out to determine if RACK-1 interacts with UNC-115/abLIM and the Rac GTPases in axon pathfinding. To analyze genetic interactions between these molecules, we used the PDE neurons, which are located at the post-deiridic region of the animal. These neurons are a good model for axon pathfinding since the reporter construct *osm-6::gfp* is expressed only in the PDEs in the post-deirid [25], [47], allowing unambiguous identification and scoring of the simple PDE axon morphology. Furthermore, the defects in PDE axon pathfinding in single mutants were weak, allowing for discrimination of genetic interactions in double mutants.

**Figure 6:**
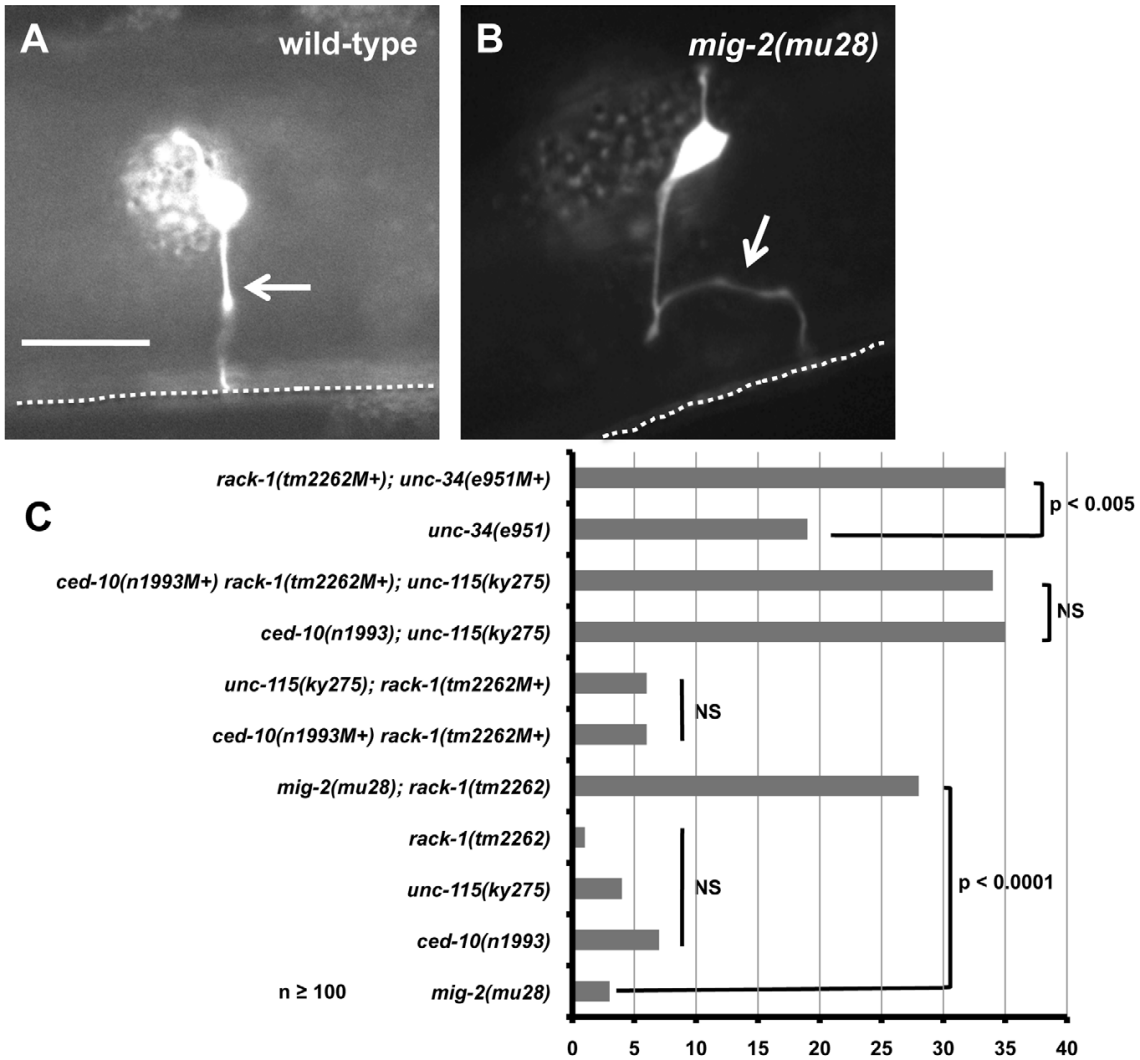
RACK-1 acts genetically in the CED-10/Rac and UNC-115/abLIM pathway. (A and B) Micrographs of animals with *osm-6::gfp* expression in the PDE neurons. The arrow in A indicates a wild-type axon, and the arrow in B indicates a misguided axon in a *mig-2(mu28)* mutant. The scale bar represents 5 µm for A and B. C) The graph represents percent of PDE axon pathfinding defects in different genotypes. At least 100 neurons were scored for each genotype, and p-value significance was determined by Fisher Exact Analysis. The genotypes indicated with an NS are not significantly different in any pairwise combination.

In wild type, the PDE cell body extends an axonal projection toward the ventral nerve cord in a straight line, where the axon then branches and extends anteriorly and posteriorly (Figure 6A) [43]. Pathfinding defects were defined as axons that were prematurely terminated or that wandered at a greater than 45 degree angle relative to the normal PDE axon (for example, Figure 6B). As shown previously, *mig-2(mu28)*, *ced-10(n1993)*, and *unc-115(ky275)*, alone had low-penetrance defects in PDE axon pathfinding on their own (3%–7%; Figure 6C). We found that *rack-1(tm2262)* also had very few defects in PDE axon pathfinding (1%; Figure 6C).

Previous results show that CED-10/Rac and MIG-2/RhoG act redundantly in PDE axon pathfinding, and UNC-115/abLIM works downstream of CED-10/Rac, in parallel to MIG-2/RhoG in PDE pathfinding [25], [48]. If RACK-1 works in the same pathway as UNC-115/abLIM, we expect that loss of function of both *rack-1* and *unc-115* would be no more severe than either mutant alone. Indeed, *rack-1(tm2262M+); unc-115(ky275)* double mutants (M+ denotes that the homozygous animal was derived from a balanced heterozygote and has wild-type maternal contribution) displayed levels of PDE axon pathfinding defects (6%; Figure 6C) that were not significantly different from *unc-115(ky275)* and *rack-1(tm2262)* alone, suggesting that UNC-115/abLIM and RACK-1 might act in the same pathway. In contrast, *rack-1(tm2262M+); mig-2(mu28)* double mutants showed significantly increased levels of defects compared to either single alone (28%; Figure 6C). This result demonstrates that *rack-1(tm2262)* can synergize with other mutants to cause axon defects, and that RACK-1 and MIG-2/RhoG might act in parallel pathways in axon pathfinding.

CED-10/Rac and UNC-115/abLIM have previously been shown to act in the same pathway in parallel to MIG-2/RhoG [25]. *rack-1(tm2262M+) ced-10(n1993M+)* double mutants displayed no significant increase in PDE defects compared to either single alone (6%; Figure 6C), consistent with the idea that RACK-1, CED-10/Rac, and UNC-115/abLIM act in a common pathway in parallel to MIG-2/RhoG in axon pathfinding. If this is the case, we would expect the *rack-1(tm2262M+) ced-10(n1993M+); unc-115(ky275)* triple mutant to be no more severe than any double mutant combination alone. As previously reported, *ced-10(n1993); unc-115(ky275)* double mutants were significantly more severe than either single alone (35%; Figure 6C) [25], [48]. This is likely due to the fact that CED-10/Rac also regulates the Arp2/3 complex in parallel to UNC-115/abLIM [16], [18]. *rack-1(tm2262M+) ced-10(n1993M+)* mutants did not show this interaction. Possibly, RACK-1 is not the only molecule regulating UNC-115 and has a weaker effect. In any case, the *rack-1(tm2262M+) ced-10(n1993M+); unc-115(ky275)* triple mutant was not significantly more severe than *ced-10(n1993); unc-115(ky275)* alone (34% compared to 35%; Figure 6C). Taken together, these results are consistent with the idea that RACK-1, CED-10/Rac, and UNC-115/abLIM act in a common pathway in axon pathfinding in parallel to MIG-2/RhoG.

UNC-34/Enabled has been shown to act in parallel to both CED-10/Rac and MIG-2/RhoG in axon pathfinding [15]. Indeed, *rack-1(tm2262M+); unc-34(e951M+)* double mutants displayed significantly increased pathfinding defects compared to *unc-34(e951)* alone (35% compared to 19%; Figure 6C). This result indicates that RACK-1 acts in parallel to UNC-34/Enabled and is consistent with RACK-1 acting with CED-10/Rac and UNC-115/abLIM in axon pathfinding.

### *rack-1(tm2262)* partially suppressed axon pathfinding defects and ectopic lamellipodia induced by activated CED-10(G12V)

**Figure 7:**
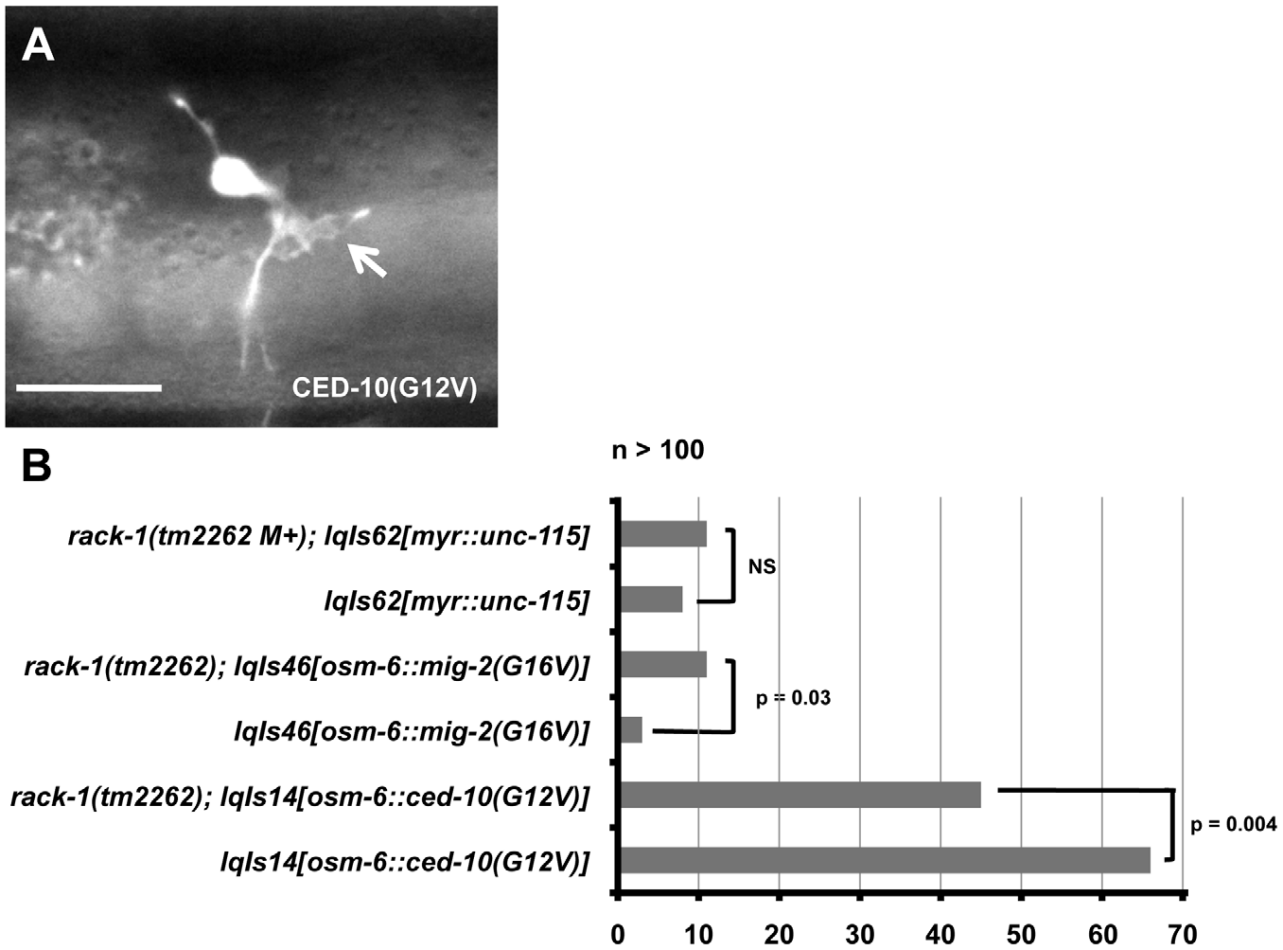
*rack-1(tm2262)* partially suppresses activated CED-10(G12V), but not MIG-2(G16V) or activated MYR::UNC-115. A) A micrograph of the PDE neuron of an adult animal expressing activated CED-10(G12V). An ectopic lamellipodial protrusion is indicated by an arrow. The scale bar represents 5 µm. B) Quantitation of PDE defects. *lqIs14* is the *osm-6::ced-10(G12V)* transgene; *lqIs46* is the *osm-6::mig-2(G16V)* transgene; and *lqIs62* is the *unc-115::myr::unc-115* transgene. At least 100 neurons were scored for each genotype, and p-value significance was determined by Fisher Exact Analysis.

Loss-of-function studies described above provide evidence that RACK-1 might act with CED-10/Rac and UNC-115/abLIM in axon pathfinding. In order to further test the relationship between RACK-1 and the Rac GTPases we next asked what effect *rack-1(tm2262)* loss of function might have on overactive Rac GTPases. Constitutively-activated Rac GTPases transgenes harbor a guanine-12-valine mutation in the GTPase binding pocket, which favors the active GTP-bound state of the GTPases. Previous studies showed that CED-10(G12V) and MIG-2(G16V) (the G12V equivalent) both caused axon pathfinding defects and drove the formation of ectopic neurites, lamellipodia, and filopodia when expressed in PDE neurons (Figure 7A), and that UNC-115/abLIM was required for ectopic lamellipodia and filopodia induced by CED-10(G12V) but not MIG-2(G16V) [25].

We determined if RACK-1 was required for the effects of CED-10(G12V) and MIG-2(G16V). CED-10(G12V) alone caused 66% of PDE neurons to have ectopic lamellipodia and filopodia in young adults (Figure 7B). *rack-1(tm2262); ced-10(G12V)* animals displayed 45% ectopic lamellipodia and filopodia, a significant reduction (p = 0.004) from CED-10(G12V) alone (Figure 7B). These data indicate that *rack-1(tm2262)* partially suppressed activated CED-10(G12V) and that functional RACK-1 might be required for the formation of ectopic lamellipodia and filopodia induced by activated CED-10. In contrast, *rack-1(tm2262)* did not suppress ectopic lamellipodia and filopodia associated with MIG-2(G16V) and in fact slightly enhanced these defects (Figure 7B; p = 0.03), indicating that this suppression is specific to CED-10(G12V). These effects are similar to those observed with the *wve-1/WAVE* mutant, which suppressed CED-10(G12V) and slightly enhanced MIG-2(G16V) [18]. These data are consistent with the idea that RACK-1 acts downstream of CED-10/Rac in parallel to MIG-2/RhoG in axon pathfinding, similar to UNC-115/abLIM.

### *rack-1(tm2262)* did not suppress defects induced by activated *myr::unc-115*

Previous studies showed that UNC-115 tagged with an N-terminal myristylation sequence (MYR) caused activation of the molecule [49]. MYR::UNC-115 localized to the plasma membrane and other membranes as expected for a myristylated protein and induced the formation of ectopic lamellipodia, filopodia and neurites in *C. elegans* neurons and in cultured mammalian fibroblasts [49]. The formation of these ectopic lamellipodia and filopodia was dependent upon the actin-binding domain of UNC-115, suggesting that the molecule was constitutively active [49].

To further dissect the interaction of RACK-1 with UNC-115, we assayed the effects of MYR::UNC-115 in a *rack-1(tm2262)* loss of function background. MYR::UNC-115 was expressed from the *unc-115* promoter (the *lqIs62* transgene), which drives expression in most neurons including PDE and the VD/DDs [49]. The *myr::unc-115* transgene scored in [49] was maintained as an extrachromosomal array. We integrated this transgene into the genome for these studies (*lqIs62)*. We found that *lqIs62[MYR::UNC-115]* caused 8% ectopic lamellipodia and filopodia in PDE neurons (Figure 7B), similar to but weaker than the extrachromosomal array effects reported in [49]. The ectopic lamellipodia and filopodia induced by MYR::UNC-115 were not significantly altered by *rack-1(tm2262)* mutation (Figure 7B), indicating that RACK-1 is not required for lamellipodia and filopodia induced by MYR::UNC-115. This result suggests that RACK-1 might act upstream of UNC-115 or together with UNC-115, or that the MYR::UNC-115 molecule acts independently of RACK-1 activity.

**Figure 8:**
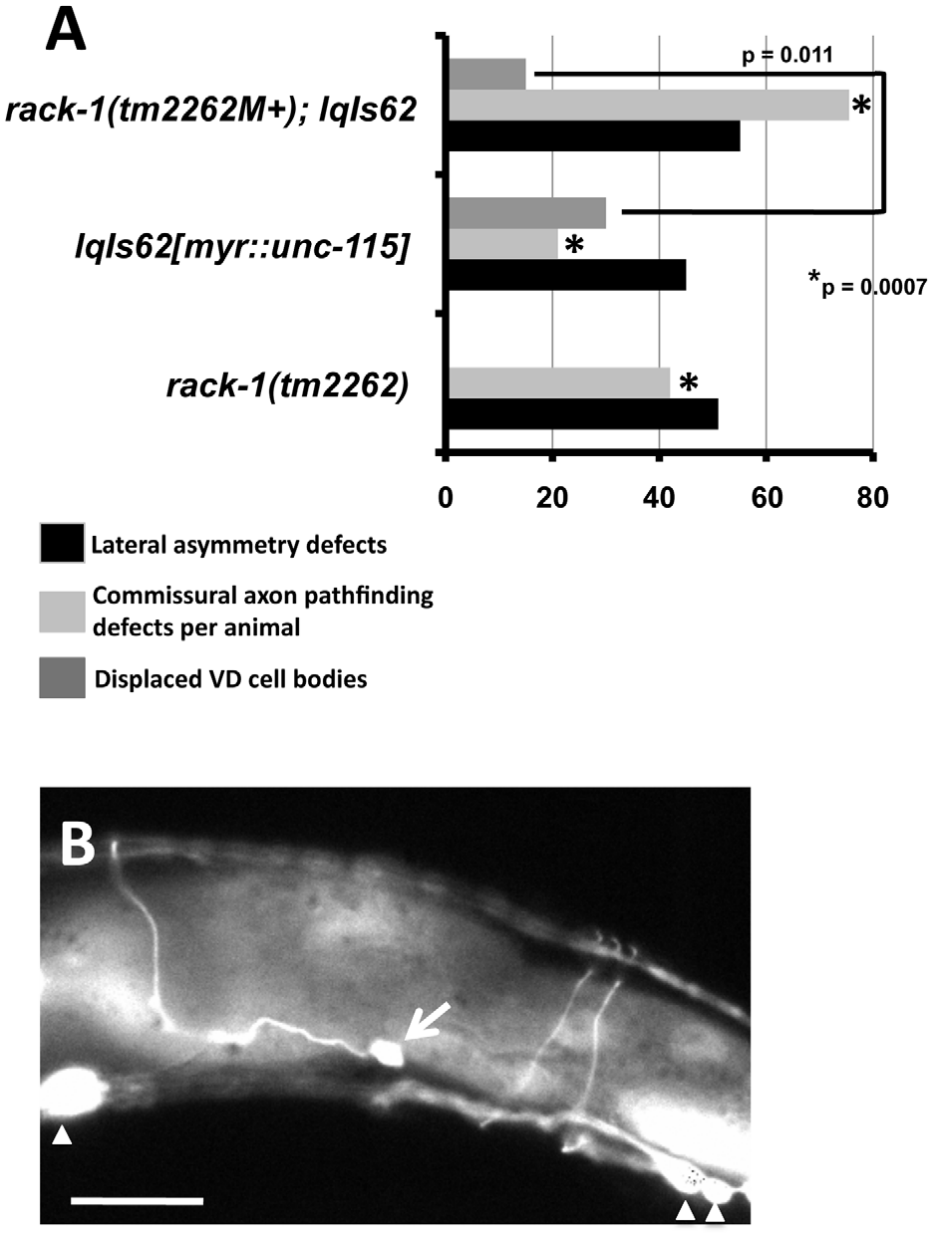
Activated MYR::UNC-115 in VD/DD motor neurons is not suppressed by *rack-1(tm2262)*. A) Quantitation of VD/DD defects caused by expression of MYR::UNC-115 from the *unc-115* promoter, which is expressed in the VD/DD neurons. The asterisks represent a test of additivity of the represented genotypes. The double mutant is significantly different from each single, and the double mutant had defects that were significantly stronger than the additive effects of each single. The following formula was used to predict the additive effect of the double mutant based on the single mutant phenotypes: *rack-1(tm2262)* + *lqIs62*− (*rack-1(tm2262)* * *lqIs62*); or 0.42+0.21− (0.42 * 0.21)  = 0.54. At least 100 animals were scored, and p-value significance was determined by Fisher Exact Analysis. B) A VD neuron cell body was laterally displaced (arrow) in a *rack-1(tm2262M+); lqIs62[myr::unc-115]* animal. *rack-1(tm2262)* partially suppressed this phenotype compared to *lqIs62* alone. Arrowheads indicate VD/DD cell bodies in their normal positions in the ventral nerve cord.

To study the interactions of *rack-1* with *myr::unc-115* in more detail, we analyzed the VD/DD motor neurons as described above. *myr::unc-115* expression caused left-right lateral asymmetry defects and commissural axon pathfinding defects as described for *rack-1(tm2262)* in Figure 2 and Figure 3 (Figure 8A). *rack-1(tm2262); myr::unc-115* animals displayed lateral asymmetry defects similar to each alone (Figure 8A; 45%–55%, not significant). This is consistent with RACK-1 acting upstream of or together with UNC-115 in the same pathway. In VD/DD commissural axon pathfinding, *rack-1(tm2262); myr::unc-115* displayed significantly increased defects compared to the additive effects of each alone (Figure 8A). Thus, *rack-1(tm2262)* might enhance *myr::unc-115* in VD/DD pathfinding.

In summary, we detected no strong suppression of *myr::unc-115* by *rack-1(tm2262)* in the PDE neurons and the VD/DD neurons. The results are consistent with RACK-1 acting upstream of or together with UNC-115 in axon pathfinding. Some context-specific interactions were observed, such as *rack-1(tm2262)* enhancing VD/DD commissural axon pathfinding, indicating that RACK-1 and UNC-115 might have distinct interactions in different contexts or developmental events.

### UNC-115 is required for the ectopic lamellipodia induced by MYR::RACK-1

**Figure 9:**
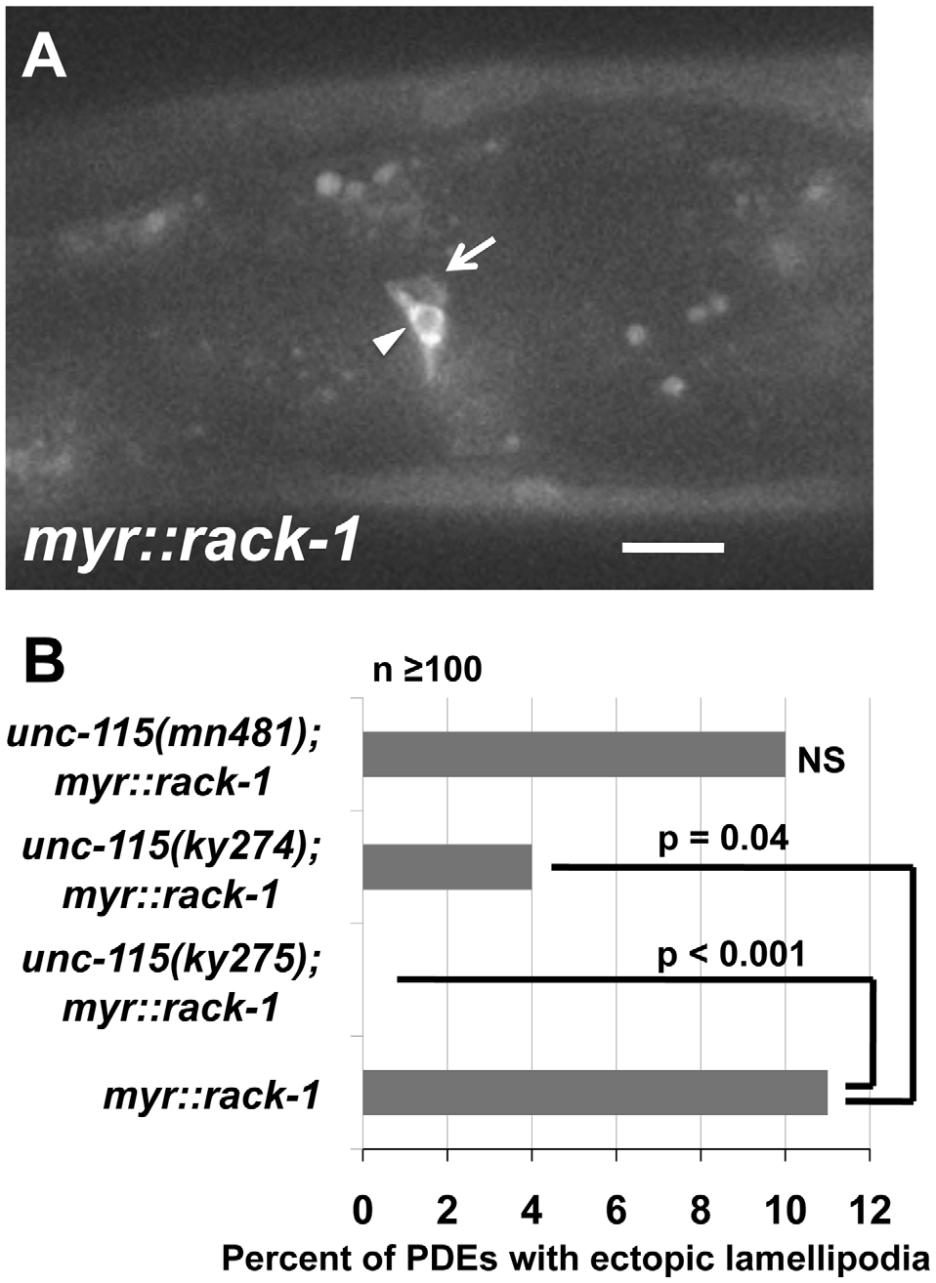
UNC-115 is required for the effects of MYR::RACK-1. A) A micrograph of MYR::RACK-1::GFP expression in the PDE neuron driven by the *osm-6* promoter. The arrowhead points to the cell surface accumulation of MYR::RACK-1::GFP, and the arrow points to an ectopic lamellipodium. The scale bar represents 5 µm. B) Quantitation of ectopic lamellipodial defects in PDEs expressing MYR::RACK-1::GFP. At least 100 animals were scored for each genotype, and p-value significance was determined by Fisher Exact Analysis.

The above results indicate that RACK-1 is not required for the effects of MYR::UNC-115, suggesting that RACK-1 might act upstream of UNC-115. To test this idea, we constructed a myristylated version of RACK-1, similar to MYR::UNC-115. We reasoned that constitutive membrane localization might activate RACK-1 as it does UNC-115. *myr::rack-1::gfp* was expressed in the PDE neurons by the *osm-6* promoter. MYR::RACK-1::GFP displayed a membrane-associated distribution (arrowhead in Figure 9A), as did MYR::UNC-115 [49]. MYR::RACK-1 animals displayed ectopic lamellipodial protrusions along the cell body, dendrite, and axon, similar to MYR::UNC-115 and activated Rac GTPases (arrow in Figure 9A). The putative null *unc-115* alleles *ky275* and *ky274* suppressed this effect (11% in *myr::rack-1* reduced to 0% and 4% in *unc-115(ky275); myr::rack-1* and *unc-115(ky274); myr::rack-1*, respectively) (Figure 9B). The hypomorphic *unc-115(mn481)* allele [17], which retains some UNC-115 activity, did not suppress *myr::rack-1*, indicating that possibly only a small amount of UNC-115 activity is required for MYR::RACK-1 to drive ectopic lamellipodia. These studies support the model that RACK-1 acts upstream of UNC-115 in lamellipodia formation.

Despite the strong genetic interactions of *rack-1* and *unc-115*, we could detect no change in distribution of UNC-115::GFP in loss of function *rack-1(tm2262)* or in the activated *myr::unc-115* transgenics (data not shown). In each case, UNC-115::GFP was present uniformly throughout the cytoplasm, similar to wild-type animals, and showed no membrane localization.

### *rack-1(tm2262)* suppresses *myr::unc-115* in VD cell body position

Activated *myr::unc-115* caused lateral displacement of GABAergic motor neuron cell bodies such that they often were found outside of the ventral nerve cord (Figure 8B). The VD GABAergic neurons are descendants of the P cells. The P cells are born laterally and the P nuclei migrate ventrally to the ventral nerve cord, where the P cells divide to produce ventral hypodermal cells including the vulva and the ventral VD neurons [50]–[51]. Failure of the ventral migration of the P nuclei can result in laterally displaced VD neuron cell bodies. This phenotype is observed in *mig-2; ced-10* double mutants, but not in *unc-115* mutants [48]. Possibly, ectopic activity from MYR::UNC-115 impedes P nucleus migration. *rack-1(tm2262)* suppressed the displaced VD cell body defect of *myr::unc-115* (Figure 8A): 30% of *myr::unc-115* animals had misplaced VD cell bodies compared to 15% of *rack-1(tm2262); myr::unc-115* (p = 0.011). This result suggests that RACK-1 might act downstream of or participate with MYR::UNC-115 in impeding P nucleus migration, again suggesting context-dependent interactions of UNC-115 and RACK-1.

### *rack-1(tm2262)* caused gonadal distal tip cell migration defects

**Figure 10:**
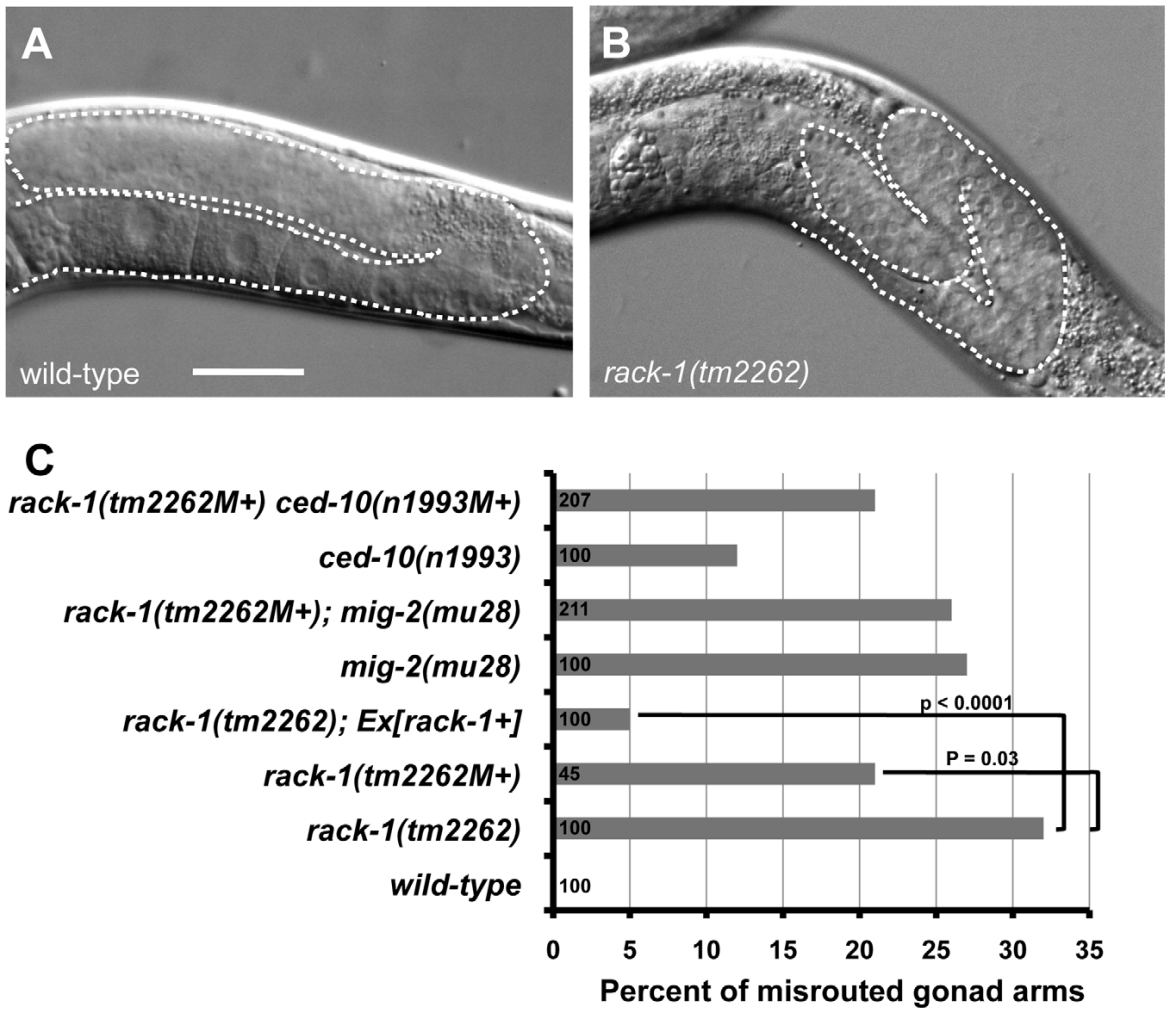
RACK-1 is required for gonadal distal tip cell migration. (A and B) Differential Interference Contrast micrographs of wild type and *rack-1(tm2262)* gonads. Dashed lines trace the outline of the gonad arm. The scale bar in A represents 10 µm. C) Quantitation of gonad arm migration defects. Number of gonad arms scored is indicated in the bars (>100). P-value significance was determined by Fisher Exact Analysis. The predicted additive effect of the *rack-1(tm2262M+) ced-10(n1993M+)* double if they did not interact was calculated by the formula *rack-1(tm2262)* + *ced-10(n1993)* − (*rack-1(tm2262)* * *ced-10(n1993*); or 0.32+0.14− (0.32 * 0.14)  = 0.42. This number was also significantly different from the observed *rack-1(tm2262M+) ced-10(n1993M+)* double mutant.

The *C. elegans* gonad is derived from two somatic cells (Z1 and Z4) surrounding the two germ cells (Z2 and Z3) [52]. Z1 and Z4 divide to produce the somatic cells of the gonad. Before morphogenesis, the gonad is oval shaped and located ventrally in the middle of the animal. The distal tip cells (DTCs) at the anterior and posterior tips of the gonad begin migration, and as they migrate they lead the gonad behind them. The DTCs migrate anteriorly and posteriorly, turn dorsally and migrate to the dorsal region of the animal, and then migrate posteriorly and anteriorly back toward the middle of the animal. DTC migration results in the U-shaped bi-lobed gonad of *C. elegans* (Figure 10A). If DTC migration is perturbed, misrouted and misshapen gonads result. The gonads of 32% of *rack-1(tm2262)* animals were misrouted (Figure 10B and 10C). Misrouting defects included failure to turn dorsally as well as extra turns, such as turning back ventrally after the dorsal migration. We did not observe gonads that had extended past their normal stopping point near the middle of the animal, as has been observed in other mutations that affect DTC migration [53], [54]. A *rack-1::gfp* transgene rescued gonad misrouting defects in *rack-1(tm2262)* (32% to 5%; p<0.005) (Figure 10C), indicating that the gonad defects were due to *rack-1* perturbation. It should be noted that *rack-1(tm2262)* homozygotes from a heterozygous mother *(rack-1(tm2262M+))* had less severe DTC migration defects compared to the *rack-1(tm2262)* animals without maternal contribution (21% compared to 32%; p = 0.03). This indicates that DTC migration defects were partially rescued by wild-type maternal *rack-1(+)* activity. *unc-115* mutants displayed no defects in DTC migration, and the gonad defects of *rack-1* were not affected by *unc-115* (data not shown). *rack-1::myc* also rescued the DTC migration defects of *rack-1(tm2262)* (data not shown). Thus, DTC migration is controlled by RACK-1 and is independent of UNC-115/abLIM.

CED-10/Rac and MIG-2/RhoG have previously been shown to control gonad distal tip cell migration [48], and we have shown here that RACK-1 also controls DTC migration. To determine if RACK-1 interacts with Rac signaling in DTC migration, we analyzed DTC migration in double mutants.

As reported previously, both *ced-10(n1993)* and *mig-2(mu28)* mutations caused defects in DTC migration (27% for *mig-2(mu28)*, 12% for *ced-10(n1993)*) (Figure 10C) [48]. We found that double mutants of *ced-10* and *mig-2* with *rack-1* showed no significant difference in defects compared to the stronger singles alone. *rack-1(tm2262M+); mig-2(mu28)* showed no significant difference (26%) compared to *mig-2(mu28)* (27%), and *rack-1(tm2262M+) ced-10(n1993)* showed no significant difference compared to *rack-1(tm2262M+)* (21% in each case).

CED-10/Rac and MIG-2/RhoG interact differently in the DTCs than they do in other tissues such as axons, in which they act in parallel redundant pathways. *ced-10; mig-2* double mutants did not display enhanced DTC migration defects compared to either single alone [48], suggesting that they might act in the same pathway or in independent pathways that each control a distinct aspect of DTC migration. Our results suggest the same for RACK-1, that it might act in a pathway independent of CED-10 and MIG-2, or that CED-10, MIG-2 and RACK-1 might all act in the same pathway in DTC migration.

## Discussion

In summary, we have presented data indicating that RACK-1 is required cell autonomously for axon pathfinding, and that RACK-1 is required for migration of the distal tip cells of the gonad. We show that RACK-1 interacts physically with UNC-115/abLIM, and that RACK-1 and UNC-115/abLIM might act in the same pathway in axon pathfinding. Consistent with this idea, RACK-1 was required for ectopic lamellipodia and filopodia induced by the activated CED-10/Rac GTPase, similar to UNC-115/abLIM. RACK-1-like molecules have been implicated in a wide variety of cellular events and have been shown to interact with a large number of distinct protein complexes, consistent with the idea of RACK molecules as scaffolds and integrators [32]. RACK molecules have been shown to control cell adhesion and migration and interact with Src, c-Abl, Rho GTPase regulators, and integrins in these events [31], [33], [39]. Our studies here suggest that RACK-1 interacts with the actin-binding protein UNC-115/abLIM and Rac GTPases in the control of axon pathfinding and cell migration.

Previous studies showed that in *C. elegans*, RACK-1 depletion by RNAi resulted in embryos with cytokinesis defects, including shorter astral microtubules, defects in chromosome separation, and defects in membrane organization and recycling endosome distribution [27]. We found that the deletion *rack-1(tm2262)* was very sick and slow growing, and gave very few progeny. *rack-1(tm2262)* produced very few embryos, suggesting that the animals had defects in sperm and/or oocyte production. Indeed, *rack-1* RNAi resulted in defects in germline membrane organization [27], consistent with the sterility that we observed in the *rack-1(tm2262)* mutant.

### RACK-1 is cell-autonomously required for axon pathfinding

*rack-1(tm2262)* mutants displayed a variety of axon pathfinding defects, including left-right choice and guidance defects of the VD and DD commissural motor axons and guidance defects of the PDE axons. VD/DD axons are dorsally directed, and PDE axons are ventrally directed, indicating that *rack-1* is not specific for any particular guidance direction. VD/DD axon pathfinding defects were rescued when *rack-1(+)* was expressed under a promoter that specifically drives expression in the GABAergic neurons including VD/DD and nowhere else, demonstrating that RACK-1 is required cell-autonomously for axon pathfinding. As RACK-1 is likely involved in many different developmental events, this result shows that the effects of RACK-1 on axon pathfinding are due to defects in the neuron itself and not a substrate or guidepost tissue such as the hypodermis or other neurons. Indeed, RACK-1 was expressed in neurons, and functional RACK-1::GFP fusion protein accumulated in the growth cones of neurons, consistent with a role of RACK-1 in growth cone cytoskeletal regulation. RACK-1::GFP also accumulated in the cell bodies and axons of neurons.

### RACK-1 controls distal tip cell migration

We have shown that *rack-1(tm2262)* mutants display defects in the structure of the gonad arms consistent with a defect in distal tip cell migration. *rack-1* is expressed in the migrating distal tip cells. The Rac GTPases CED-10/Rac and MIG-2/RhoG also each affect distal tip cell migration, but do not show the phenotypic synergy in DTC migration as is observed in axon pathfinding [48]. Thus, CED-10/Rac and MIG-2/RhoG might act in independent pathways that control distinct aspects of DTC migration.

DTC migration defects in *mig-2; rack-1* and *ced-10 rack-1* double mutants were not significantly different than the stronger single mutants alone. This result suggests that RACK-1 might act in a pathway independent of MIG-2 and CED-10, or that all three act in a common pathway. This again points to context dependent differences in the function of RACK-1 and suggests that RACK-1 might interact with different effectors in different ways in different cells and cellular events. Indeed, the effect of RACK-1 on DTC migration is likely to be independent of UNC-115/abLIM, as *unc-115* mutants have no effect on DTC migration alone or in any double mutant combination analyzed so far, including *ced-10* and *mig-2*.

### RACK-1 acts in the CED-10/Rac and UNC-115/abLIM pathway in axon pathfinding

A model of RACK-1 interaction with CED-10/Rac and UNC-115/abLIM is shown in Figure 10A. Double mutant analysis showed that *rack-1(tm2262)* synergized with *mig-2/RhoG* and *unc-34/Enabled* in PDE axon pathfinding, similar to *unc-115/abLIM* and *ced-10/Rac*. *rack-1(tm2262)* did not synergize with *unc-115/abLIM* or *ced-10/Rac*, consistent with the idea that they act in the same pathway in parallel to *mig-2/RhoG* and *unc-34/Enabled*.

Activated CED-10(G12V) drives the formation of ectopic lamellipodia and filopodia in PDE neurons, and *unc-115* loss of function suppresses this effect [25]. We show here that *rack-1(tm2262)* also partially suppressed ectopic lamellipodia and filopodia caused by CED-10(G12V), indicating that RACK-1 is required downstream of CED-10/Rac in lamellipodia and filopodia formation (Figure 10A). This result suggests that RACK-1 might normally be required for lamellipodia and filopodia formation. This is in contrast to the seven-WD repeat protein SWAN-1, which physically interacts with the UNC-115 LIM domains and with CED-10/Rac but which is normally required to inhibit CED-10/Rac signaling in lamellipodia and filopodia formation [26]. Thus, these two seven-WD repeat proteins SWAN-1 and RACK-1 might have opposite effect on CED-10/Rac signaling: SWAN-1 inhibits it, and RACK-1 is required downstream of it to form lamellipodia and filopodia. That RACK-1 is required for lamellipodia and filopodia formation downstream of CED-10/Rac suggests that RACK-1 might be acting directly in cytoskeletal regulation. It is also possible that RACK-1 exerts its effects downstream of Rac GTPases through transcriptional or translational control, but the fact that RACK-1 interacts physically with the actin-binding protein UNC-115/abLIM supports the idea that RACK-1 directly controls cytoskeletal signaling.

**Figure 11:**
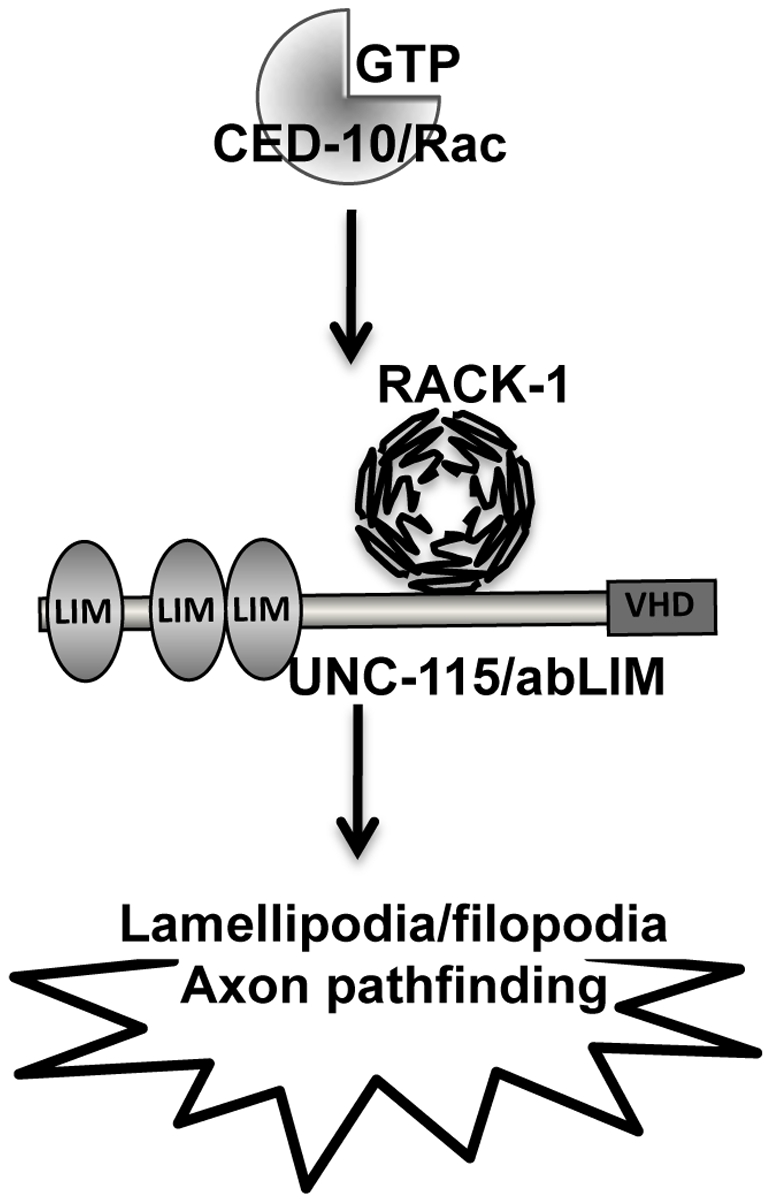
RACK-1 might regulate UNC-115/abLIM downstream of CED-10/Rac in lamellipodia and filopodia formation. A linear pathway representing the genetic interactions between *ced-10/Rac, rack-1*, and *unc-115*. RACK-1 acts downstream of CED-10/Rac and controls UNC-115/abLIM. LIM  =  LIM domain; VHD  =  villin headpiece domain.

RACK-1 physically interacts with UNC-115/abLIM and genetically acts in the same pathway in axon pathfinding. UNC-115 can be activated constitutively by the addition of an N-terminal myristylation sequence [25], which mediates the covalent attachment of a fatty acid myristyl residue to the protein and drives localization to membranes, including the plasma membrane. MYR::UNC-115 also drives the formation of ectopic lamellipodia and filopodia, similar to but weaker than CED-10(G12V) [25]. No strong suppression or enhancement of axon pathfinding defects were observed in double mutants of *rack-1(tm2262)* and *myr::unc-115*. One interpretation of these data is that RACK-1 does not act downstream of UNC-115/abLIM and instead might act together with or upstream of UNC-115/abLIM. Indeed, *unc-115* mutations suppressed the ectopic lamellipodia caused by MYR::RACK-1, indicating that UNC-115 acts downstream of RACK-1. These results are consistent with a model in which RACK-1 acts downstream of CED-10/Rac and upstream of UNC-115/abLIM in axon pathfinding (Figure 11).

### RACK-1 and MYR::UNC-115 display context-specific interactions

RACK-1 and UNC-115 displayed context-dependent interactions in addition to those described in the PDE neurons above. First, *rack-1* slightly but significantly increased VD/DD commissural pathfinding defects caused by *myr::unc-115*. We do not understand the nature of the VD/DD axon pathfinding defects caused by MYR::UNC-115, but it is possible that they are due to excessive lamellipodial and filopodial protrusion, possibly in the growth cone. If this is the case, these effects were enhanced by *rack-1* loss of function, suggesting that RACK-1 might negatively regulate MYR::UNC-115 in this context, possibly by excluding MYR::UNC-115 from regions in which it induces lamellipodia and filopodia.

Second, *rack-1(tm2262)* suppressed the lateral displacement of VD cell bodies caused by *myr::unc-115*. Laterally misplaced VD cell bodies are indicative of a defect in the ventral migration of the nuclei of the P cells. UNC-115 is not normally involved in P nucleus migration, but *ced-10/Rac* and *mig-2/RhoG* act redundantly in the process [48]. Possibly, *myr::unc-15* ectopically interferes with P nucleus migration, and RACK-1 is required for this effect. In this case RACK-1 might normally positively regulate MYR::UNC-115. In any case, these data indicate that RACK-1 and UNC-115 might have distinct interactions in different cellular contexts.

In summary, these studies suggest that RACK-1 acts in a common pathway with CED-10/Rac and UNC-115/abLIM in axon pathfinding (Figure 11). These studies implicate the Receptor of Activated C Kinase as a new Rac GTPase effector molecule, as RACK-1 acts downstream of CED-10 and upstream of UNC-115/abLIM in axon pathfinding. Future studies will be directed at understanding the roles of plasma membrane localization and phosphorylation in the regulation of this pathway.

## Materials and Methods

### *C. elegans* genetics and culture

*C. elegans* culture and techniques were performed using standard protocols [55]–[56]. All experiments were performed at 20°C. The *rack-1(tm2262)* allele was provided to us by the National Bioresource Project for the Experimental Animal “Nematode C. elegans” (S. Mitani), and was outcrossed to wild-type N2 animals three times before analysis. Polymerase chain reaction (PCR) was used to verify the homozygosity of *rack-1(tm2262)* in strains. The following mutations and genetic constructs were used: *LGII: juIs76[unc-25::gfp]; LGIV: ced-10(n1993), rack-1(tm2262), nT1 IV:V, lqIs3[osm-6::gfp]; LGV: unc-34(e951); LGX: unc-115(ky275), mig-2(mu28), lqIs2[osm-6::gfp]; LG?: lqIs62[myr::unc-115(+)]. C. elegans* transformation was performed by standard techniques using DNA microinjection into the syncytial germline of hermaphrodites [57]. Transgenes were integrated into the genome using trimethylpsoralen and standard techniques [58]–[59].

All micrographs were obtained on a Leica DMRE microscope with a Qimaging Rolera MGi EMCCD camera or a Qimaging Retiga CCD camera. Openlab and IPlab software were used to obtain images.

### Molecular biology

All coding regions amplified by PCR were sequenced to ensure the absence of mutations in the sequence. PCR, recombinant DNA and other molecular biology techniques were performed according to standard techniques [60]. Primer and plasmid sequences are available upon request.

### Scoring of VD, DD, and PDE axon defects

Axon pathfinding defects were scored with fluorescence microscopy of hermaphrodite animals in the fourth larval stage (L4) or young adults expressing a green fluorescent protein transgene for specific cells. To visualize and score the axons of VDs and DDs, animals harboring an *unc-25 promoter::gfp* integrated transgene (*juIs76 II*) were used [45]. To visualize and score PDE axons, animals harboring an *osm-6 promoter::gfp* integrated transgene (*lqIs2 X* or *lqIs3 IV*) were used [25], [47].

#### VD/DD lateral asymmetry

VD/DD commissural axons normally extend up the right side of the animal (except the VD1/DD2 commissure, which extends on the left). The number of animals with aberrant left-side extension of commissural axons was scored.

#### VD/DD axon pathfinding

A VD/DD commissural axon that failed to reach the dorsal nerve cord or that wandered laterally before reaching the dorsal nerve cord was considered mutant. The percent of animals with pathfinding defects was noted, and the percentage of defective axons was noted.

#### VD cell body displacement

The VD neurons are descendants o the P cells. If the P nuclei fail to migrate ventrally, the resulting VD cell bodies can be laterally displaced out of the ventral nerve cord. The percentage of animals with laterally displaced VD cell bodies was scored.

#### PDE axon pathfinding

The cell bodies of the PDE neurons (PDEL and PDER) are situated in the posterior lateral post-deirid ganglion. PDEs extend an axon ventrally to the ventral nerve cord, which then bifurcates and extends anteriorly and posteriorly in the ventral nerve cord. If the axon failed to reach the ventral nerve cord or wandered beyond a 45° angle from a straight line ventrally from the cell body, it was considered mutant. Significance was determined using Fisher Exact Analysis.

### Activated *ced-10(G12V)*, *mig-2(G16V)*, and *myr::unc-115* transgenes

*ced-10(G12V)* and *mig-2(G16V)* transgenes under the control of the *osm-6* promoter were used as described previously [25]. A *myr::unc-115* transgene under the control of the *unc-115* promoter was used as described previously [49].

### Scoring of distal tip cell migration defects

Gonadal distal tip cell migration defects were scored by Differential Interference Microscopy in young adult hermaphrodite animals. Any deviation from the normal U-shape of gonad arms was scored as defective, including failure to migrate fully, failure to make a dorsal turn, failure to make an anterior or posterior turn, or extra dorsal-ventral or anterior-posterior turns. Significances of differences (p values) were determined using Fisher Exact Analysis.

### RACK-1 transgenes

A full-length *rack-1(+)* transgene was generated by PCR from genomic DNA (based on the Wormbase gene model K07D7.1) and included the entire upstream *rack-1* region (∼2.5 kb), the coding region, and the downstream region past the poly-A addition site (Figure 1E). *rack-1::gfp* and *rack-1::myc* fusion constructs were generated by amplifying the entire *rack-1* upstream region and coding region lacking the stop codon fused in frame to *gfp* or *myc*. The *unc-25 promoter::rack-1::gfp* fusion protein was generated by amplifying the *rack-1* coding region lacking the upstream region. This fragment was placed downstream of the *unc-25* promoter and fused in frame to *gfp* at the 3′ end.

### UNC-115 yeast two-hybrid screen

The two-hybrid screen was conducted at the Molecular Interaction Facility at the University of Wisconsin-Madison (thanks to E. Maher). In a liquid multi-well format, approximately 36 million *C. elegans* cDNA clones representing both oligo dT and random-primed libraries were screened via mating. UNC-115 was fused to the GAL4 DNA-binding domain in the pBUTE plasmid and the prey cDNAs were fused to the GAL4 activation domain in the pACT plasmid. In the yeast strain, the bacterial *lacZ* gene and the *HIS5* gene were under the control of a GAL4-regulated promoter. The interaction screen consisted of assaying β-galactosidase (β-gal) activity (for *lacZ)* and growth on 25 mM 3-aminotriazole (3-AT) (for *HIS5)*. This analysis identified 244 potential interacting cDNAs that had β-gal activity and grew on 25 mM 3-AT. From these 244, 142 isolates activated both *lacZ* and *HIS5* similarly when re-tested. Of these, 124 were bait-specific and did not activate when the bait plasmid was removed. These cDNAs were sequenced, and seven of these were found to represent the K07D7.1 gene in Wormbase (*rack-1)* [27].

### RACK-1::MYC immunoprecipitation

In order to obtain large amounts of *C. elegans* protein extract, animals carrying an integrated *rack-1::myc* transgene were raised at room temperature in a liquid culture containing 2.5 mg cholesterol, 0.05 mg/mL streptomycin, *Escherichia coli* strain HB101 and M9 buffer (up to 500 mL). After about a week, these animals were harvested and snap-frozen in liquid nitrogen. We then added lysis buffer (1× PBS, 10% glycerol, 0.1% NP40, 0.1% Tween) in a 1∶1 ratio, and then 1 mM of phenylmethanesulphonylfluoride. We lysed the animals with glass beads in a beater for two cycles of 1 minute each. The supernatant was then collected and stored at −80°C for further experiments. We based our immunoprecipitation assays in Clonetech Laboratories' protocol No. PT3407-1 (Clonetech). We performed the standard immunoprecipitation assays as described in [26] using protein G (Zymed) and anti-Myc monoclonal antibody (Clontech). The anti-UNC-115 antibody is described in [26].
